# Non-transcriptional processes in circadian rhythm generation

**DOI:** 10.1016/j.cophys.2018.10.003

**Published:** 2018-10

**Authors:** David CS Wong, John S O’Neill

**Affiliations:** MRC Laboratory of Molecular Biology, Francis Crick Avenue, Cambridge, CB2 0QH, UK

## Abstract

‘Biological clocks’ orchestrate mammalian biology to a daily rhythm. Whilst ‘clock gene’ transcriptional circuits impart rhythmic regulation to myriad cellular systems, our picture of the biochemical mechanisms that determine their circadian (∼24 hour) period is incomplete. Here we consider the evidence supporting different models for circadian rhythm generation in mammalian cells in light of evolutionary factors. We find it plausible that the circadian timekeeping mechanism in mammalian cells is primarily protein-based, signalling biological timing information to the nucleus by the post-translational regulation of transcription factor activity, with transcriptional feedback imparting robustness to the oscillation via hysteresis. We conclude by suggesting experiments that might distinguish this model from competing paradigms.

## Highlights

• Transcriptional feedback is insufficient to account for circadian rhythm generation.
• Post-translational regulators of daily cellular clocks are conserved among eukaryotes.
• Eukaryotic circadian timekeeping may result from divergent evolution.

**Current Opinion in Physiology** 2018, **5**:117–132

This review comes from a themed issue on **Circadian rhythms**

Edited by **Martin Young**, **Karen Gamble** and **Hugh Piggins**

For a complete overview see the Issue and the Editorial

Available online 6th November 2018

**https://doi.org/10.1016/j.cophys.2018.10.003**

2468-8673/© 2018 MRC Laboratory of Molecular Biology. Published by Elsevier Ltd. This is an open access article under the CC BY license (http://creativecommons.org/licenses/by/4.0/).

## Introduction: the utility of biological timekeeping at multiple levels of organisation

Life consists of the spatiotemporal organisation of chemical processes in order to maintain homeostasis. As the Earth rotates, organisms encounter daily variations in environmental factors such as light, temperature and food availability. Most organisms have developed internal daily timing systems that allow them to reliably anticipate and adapt to these external cycles, conferring a competitive advantage over organisms that do not [1, 2, 3, 4, 5, 6, 7]. The persistence of circadian (about daily) rhythms when organisms are removed from external stimuli demonstrates their endogenous nature.

The history of circadian research begins with Jean-Jacques de Mairan’s fascinating observation in 1729 [8], that the leaves of *Mimosa pudica* open and close on a daily basis, and that this biological rhythm persists in constant darkness; this shows that the behaviour is under the control of an endogenous circadian timekeeping mechanism — a daily ‘biological clock’. Subsequently the circadian organisation of very many biological activities has been observed, from the eclosion activity of *Drosophila pseudoobscura* to the wheel running activity of *Mus musculus* [8]. Colin Pittendrigh, a founding figure in chronobiology [9], laid out a set of ‘Empirical Generalisations’ at the 1960 Cold Spring Harbour Symposium on Quantitative Biology [10] that define the fundamental characteristics of a circadian rhythm: 1) An endogenous oscillation with an approximately 24 hour period under constant conditions; 2) Period length is temperature-compensated (temperature coefficient, Q_10_, of 0.9–1.2); 3) The phase of oscillation is entrained by (i.e. synchronised with) environmental stimuli that themselves normally change with 24-hour periodicity.

In mammals, the hypothalamic suprachiasmatic nucleus (SCN) acts as ‘master pacemaker’, coordinating the circadian organisation of behaviour, physiology and cellular functions by neuronal and hormonal means [11, 12, 13, 14, 15, 16, 17, 18]. However, it has been clear for more than 10 years that the fundamental unit of circadian timekeeping is the cell, since circadian regulation of gene expression and cellular activity persists *ex vivo* in almost all mammalian cell types tested [19, 20, 21, 22]. Data from mouse models show that homozygous mutations that impair cell-autonomous circadian regulation of transcription in cultured cells also impair circadian organisation at higher levels of organismal biology [23,24,25^•^,^26^]. This suggests that circadian organisation of rest-activity cycles and overt physiology is driven by the rhythmic regulation of gene expression on a cellular level.

Cell-autonomous circadian timekeeping also extends to the green and fungal eukaryotic lineages [27]. Thus, whilst the translational and economic consequences of circadian rhythms and their disruption have been primarily studied in multicellular organisms, it is necessary to look towards the molecular biology of the cell when considering the fundamental mechanisms that drive this 24-hour regulation at multiple levels of biological scale. This review will therefore focus on the molecular determinants of cell-autonomous circadian timekeeping.

## Transcription/translation feedback facilitates rhythmic gene expression

In healthy young wild type mice, 43% of protein-encoding genes in the genome are subject to circadian regulation in at least one tissue [28], with available data from humans suggesting that similar proportions of our own genomes are subject to diurnal regulation in healthy individuals [29^•^]. Circadian regulation of gene expression in mammalian and other eukaryotic cells has been proposed to rely upon delayed transcriptional feedback repression at a handful of genomic loci (so-called ‘clock genes’), which auto-regulate their own expression, as well as that of many ‘clock-controlled genes’ that vary between tissues [27,30,31]. From the context in which it is most often used, the term ‘clock gene’ generally refers to a gene whose expression contributes to the fidelity of circadian timekeeping, and whose gene product directly or indirectly represses its own activity to elicit a ∼24 hour oscillation in its abundance and/or activity [32]. The clock gene circuitry that facilitates these delayed transcriptional-translational feedback loops (TTFLs) has been successfully delineated for several model organisms within each eukaryotic kingdom [27,33], with current understanding of the mammalian TTFL summarised in an excellent recent review [34^•^]. Whilst in general there exists a fairly poor correlation between the identities, relative amplitude and waveform of rhythmically abundant mRNA transcripts compared with their encoded proteins [35, 36, 37, 38, 39, 40], daily oscillations in clock gene transcription occur in all mammalian tissues *in vivo* [28]. Combined with the strong phenotype of clock gene loss or gain-of-function mutations therefore, there exists overwhelming evidence for the idea that temporal coordination of gene expression programs by the cycling activity of clock proteins is the ultimate means by which cellular clocks organise most daily biological functions [41]. To provide context to the non-transcriptional aspects of circadian timekeeping, we will briefly describe the main TTFL features that facilitate circadian gene expression rhythms in mammalian cells.

The transcription factors BMAL1 and CLOCK are heterodimeric partners, belonging to the basic helix-loop-helix (bHLH) – PER-ARNT-SIM (PAS) transcription factor family [42, 43, 44]. Whilst CLOCK functions semi-redundantly with NPAS2, the recruitment of BMAL1-containing complexes to promoter/enhancer regions of the *Period* genes (encoding transcriptional repressors PER1, 2, 3) and *Cryptochrome* genes (encoding obligate co-repressors CRY1, 2) appears to be essential for their differential expression over the daily cycle. PER and CRY proteins assemble into large macromolecular complexes to mediate the repressive limb of the feedback loop, rhythmically binding to and inhibiting the transcriptional activity of BMAL1-containing complexes [45^•^,^46^,^47^,^48^]. This represses transcription from their own genomic loci as well as many clock-controlled genes [49,50]. In recent years it has become clear that this model is much simpler than the reality, in that each ‘clock gene’ has many additional functions beyond circadian regulation [51,52^•^,^53^,^54^, ^55^, ^56^, ^57^, ^58^]. Moreover the activating and repressive transcriptional complexes do not operate in isolation, rather they function in tandem with enhancer elements to effect the differential recruitment of assorted histone modifying and chromatin remodelling complexes, resulting in large-scale tissue-specific changes in chromatin compaction and accessibility [34^•^,^50^,^59^,60^•^,61^•^].

**Figure 1:**
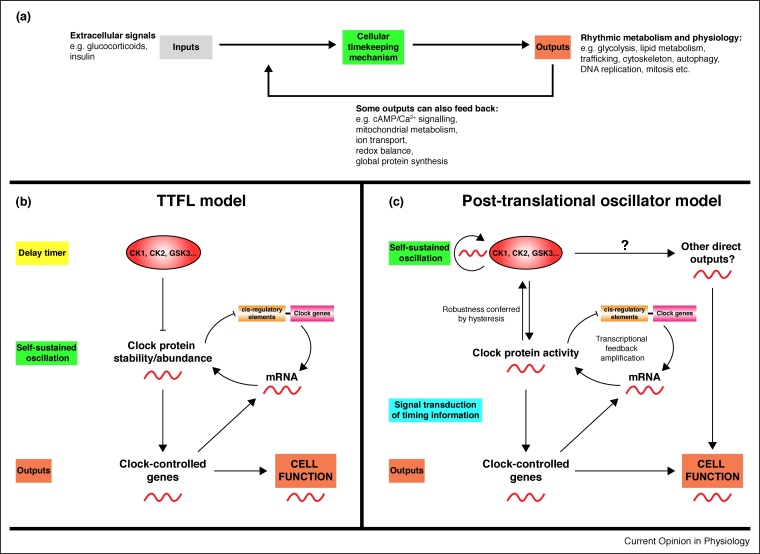
**Models for circadian rhythm generation**. **(a)** Biological clocks consist of a timekeeping mechanism whose phase, period and amplitude may be regulated by various inputs. Outputs of the clock control rhythmic metabolism, physiology and behaviour of organisms, and some of these may in turn feedback to modulate the timekeeping mechanism itself, thus also acting as inputs. Rhythmic input regulation is not essential for circadian rhythm generation however. **(b)** TTFL-based model for cellular circadian timekeeping: the TTFL itself can sustain oscillations, with the 24-hour period conferred by a post-translational delay-timer. The clock-controlled genes are direct outputs of the TTFL, and result in rhythmic cell function. Some of these outputs can feed back to regulate TTFL function. **(c)** Post-translational model for cellular circadian timekeeping: a self-sustained post-translational timekeeping mechanism is sufficient to sustain ∼24h rhythms in enzyme activity. The TTFL acts as a signal transducer, receiving timing information from this biochemical oscillation through post-translational modification of TTFL components, to differentially regulate transcription of clock and clock-controlled genes. The TTFL may confer robustness upon the post-translational oscillation by amplifying timing information and also by differentially regulating the activity of the enzymes that post-translationally modify clock proteins.

The broader rhythmic regulation of gene expression programs is elaborated by several auxiliary feedback mechanisms, whose relative contribution to cellular clock function likely varies between tissues [26,62]. For example, CLOCK/BMAL1 complexes activate the transcription of nuclear receptors REV-ERBα (*Nr1d1*) and REV-ERBβ (*Nr1d2*), which mediate feedback repression of *Bmal1* and other targets *via* ROR-binding promoter elements [63]. A further transcriptional feedback loop involves the activating transcription factor DBP (D-box binding protein), whose rhythmic transcription is dependent on CLOCK/BMAL1 activity [64,65]. DBP binds to D-box promoter elements, antagonising the repressor NFIL3 (nuclear factor, interleukin-3 regulated), which is regulated by REV-ERB-ROR loop activity [66,67]. These auxiliary loops integrate with the central TTFL to fine-tune the amplitude and phase of downstream clock-controlled gene expression [68], but they are not essential for rhythms of the central TTFL circuit [26]. Finally, as we will discuss later, the activities of many rhythmically regulated cellular processes (outputs) also feedback to modulate the fidelity of circadian timekeeping, further increasing the apparent complexity of the cellular clockwork (Figure 1a).

The explicit molecular mechanism that determines the 24-hour periodicity of clock gene expression cycles in mammalian cells is not understood. TTFLs are common in cell biology, and represent a common means by which cells terminate a signalling process and return to baseline. These work by the negative feedback of immediate-early gene products that induce a change in gene expression whilst simultaneously sowing the seeds for their own destruction [69]. Such gene expression feedback loops endow cell-autonomous oscillations upon the ERK signalling pathway (2–3 hour) [70,71], NF-κB pathway (3–4 hour) [72], and the developmental segmentation clock (∼1–2 hour) [73,74], with period lengths reflecting the time taken for RNA processing and translation, and oscillations that damp out after several cycles (unlike circadian oscillations) [20,75]. In the context of the TTFL whose components co-ordinate mammalian circadian gene expression rhythms, the summed time constants associated with transcriptional feedback are insufficient to account for the day-long interval between transcriptional activation of the *Per* and *Cry* genes and the disassembly of their repressive complexes. For this reason, and supported by strong genetic evidence [76, 77, 78, 79, 80, 81, 82], post-translational^1^ regulation of clock protein activity by casein kinases and other enzymes is invariably invoked to accommodate the lengthy delay that occurs with each circadian cycle (Figure 1b).

## Layers of complexity beyond transcriptional feedback loops

In mouse tissues *in vivo* [37,39,40,83,84], in SCN slices *ex vivo* [85] and in cultured fibroblasts *in vitro* [35], roughly 15% of detected proteins exhibited significant circadian abundance rhythms. Such hypothesis-free proteomic studies have revealed a surprising discrepancy between cycling proteins and their mRNAs. Almost half of cycling proteins in mouse tissues do not have a corresponding cycling transcript, as shown in liver and SCN by a two-dimensional difference gel electrophoresis (2D-DIGE) approach [39,85], and recapitulated in cultured *Drosophila* Schneider 2 (S2) cells where only 7% of detected rhythmic proteins had a corresponding cycling mRNA [86]. This indicates the major role that post-transcriptional processes must play in the regulation of proteins whose abundance oscillates over circadian timescales.

Along these lines, Robles *et al.* revealed that half of the cycling proteome was delayed by more than 6 hours with respect to the corresponding mRNA profile, and that 20% of the cycling proteome did not have correspondingly cycling transcripts [40], with others reporting similar findings under diurnal conditions [37]. Moreover, recent technical innovations in mass spectrometry have permitted the detection of time-of day variations in the post-translational modifications of purified *Neurospora* FREQUENCY (FRQ) protein [87], and in mouse liver phosphoproteome and acetylome [88^•^,^89^], with as great as 25% of the 20,000 detected phospho-sites showing circadian rhythms. In many contexts, reversible allosteric and post-translational regulation of protein activity allows more efficient regulation of cellular function than do changes in gene expression. For example, glycolytic enzymes have such high expression levels and turnover numbers that their abundance is rarely rate-limiting; and so metabolic flux is regulated primarily allosterically and by covalent modification [90,91]. Therefore, these innovative new circadian post-translational “-omics” studies all point towards a critical participation of post-translational regulation in the circadian control of cell function. Put simply, circadian transcriptional rhythms may be insufficient to fully account for the daily regulation of cellular processes.

## Discrepancies between the TTFL model and experimental observations

Indeed, it is not clear that rhythmic expression of central TTFL components is actually essential for circadian timekeeping to persist at the cellular level. For gene expression rhythms to be observed in cultured mammalian cells, all that seems to be absolutely required is that the activity of each central TTFL component BMAL1 (or paralog BMAL2), CLOCK (or paralog NPAS2), PER1 or PER2, and CRY1 or CRY2 reside within a permissive range [92,93,94,95^•^,^96^,^97^]. Consistent with this, circadian transcriptional rhythms are not observed in cells cultured from mice that are deficient for *Bmal1*, or both *Cry* genes, or both *Per1* and *Per2*. Critically though, in complementation assays, constitutive expression of BMAL1 or CRY1 or PER2 in their respective single or double knockout backgrounds is sufficient to rescue circadian gene expression rhythms [26,97,98,99^•^,^100^]. When the repressor clock proteins CRY1 or PER2 are instead-over expressed under strong constitutive promoters, in Cry- or Per-deficient backgrounds respectively, rhythmic transcription is again severely impaired [97,101]. Similarly, constitutive expression of the essential repressors (*per* or *tim)* of the central TTFL of the clock in *Drosophila melanogaster* is sufficient to facilitate circadian rhythms, whilst overexpression or knockout of either gene abolishes rhythms in behaviour [102]. In addition, recent experiments using the fungal clock model, *Neurospora crassa*, showed that circadian rhythms in the activity of FRQ (the major TTFL repressor protein in *Neurospora*, functionally equivalent to PER) could be decoupled from its stability by deletion of the F-box protein (FWD1) that normally facilitates its degradation, thus allowing FRQ to accumulate to constant levels but with persistent activity rhythms [103]. These lines of evidence support the possibility that rhythmic clock protein activity is the key determinant of circadian transcriptional oscillations, with transcriptional feedback reinforcing clock protein activity rhythms rather than generating them. Certainly, the elegant reverse genetics findings described above suggest that the critical transcription factors of circadian TTFLs simply need to be expressed within an appropriate concentration range in order for activity rhythms to be observed.

## Post-translational models for circadian rhythm generation

Following from this, if rhythmic clock protein activity, rather than abundance, is the essential prerequisite for circadian recruitment of downstream transcriptional programs, it is plausible that loss-of-function mutations in central and auxiliary TTFL components are simply epistatic to the expression of most cellular circadian rhythms [58,104,105]. In this case, an alternative model for the causal relationships underlying circadian regulation of gene expression suggests itself: that post-translational modifications of clock proteins are necessary and sufficient to confer circadian regulation to their activity (Figure 1c). Since most clock proteins auto-regulate their own production, and because any mRNA or protein with a circadian rhythm of production will exhibit rhythmic abundance if its half-life is less than 6 h [106], rhythmic clock protein activity will inevitably generate rhythms in the transcript and protein levels of most clock genes (e.g. *Per2* and PER2 half-lives are <3 hour) [107, 108, 109]. In this alternative model, rather than determining its period of oscillation, the utility of transcriptional feedback repression in the cellular circadian clock is primarily to amplify post-translationally derived oscillations in clock protein activity through oscillations in clock protein abundance, effectively increasing the ‘gain’ of the transcriptional output (Figure 1c). Amplifying rhythms in clock protein activity through rhythms in clock protein abundance confers the intrinsic advantage of increased scope for phase-dependent differential recruitment of downstream clock-controlled gene expression programmes. In other words, TTFLs are required for high amplitude clock-controlled gene expression, rather than circadian rhythm generation itself. In turn, this predicts that amino acid substitutions that affect the activity of clock proteins will have more marked effects on the amplitude and period of gene expression rhythms than those which affect their abundance, until this latter exceeds or falls below their permissive concentration range [25^•^,^103^].

Extending this argument, if circadian-regulated transcription factors, such as PER, are considered as executive signal transducers rather than rhythm generators (Figure 1c), the critical advantage of a post-translational timekeeping mechanism would be the scope for hysteresis. Hysteresis describes the phenomenon whereby the activity of regulatory complexes is a function of not only their current condition (e.g. phosphorylation status) but also their history due to changes in constituent protein abundance (i.e. due to changes in transcription/translation/degradation) [110]. This requires bi-directional regulation between TTFL and post-translational timekeeping mechanisms, and indeed the reciprocal regulation of activity between PER2 and one of its post-translational regulators, CK1ε, was recently reported [111].

**Figure 2:**
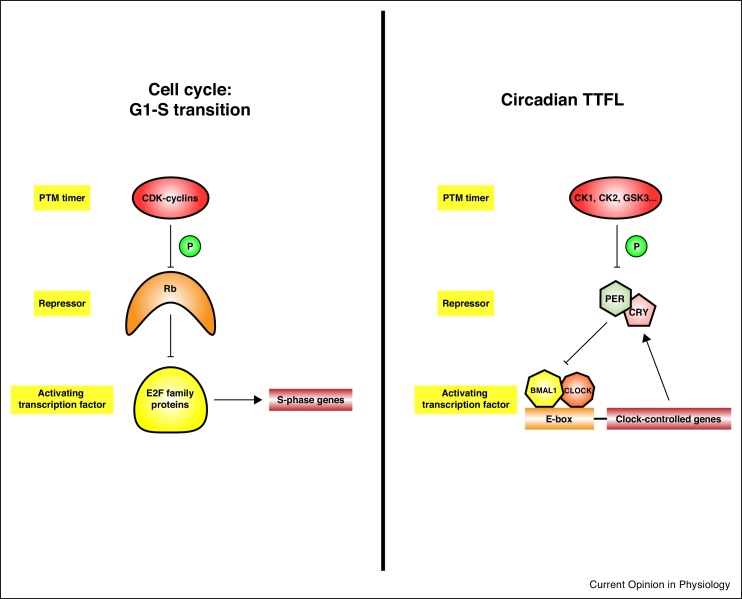
**Comparing the circadian cycle to the cell cycle G1/S transition**. A critical point for both the cell cycle and the circadian cycle is the activation of key transcription factors. This is achieved in both cases by the relief of repression by a negative regulator. The mechanism for this is phosphorylation: of Rb by cyclin-dependent kinases for the cell cycle, and of PER by CK1δ/ε for the circadian cycle. Hence the de-repression of crucial activating transcription factors via kinase activity is a common network motif for the temporal regulation of cellular processes.

This post-translational view of the cellular circadian cycle has obvious parallels with the cell division cycle transition between G1 (growth phase) and S phase (DNA replication). This transition is known as the ‘restriction point’ [112], because it is thought to be the point of no return for cell cycle entry (Figure 2). Double negative feedback regulation of transcriptional control factors (activating E2F and repressor Rb proteins) imparts bistability around the G_1_-S phase transition, such that each steady state is robustly buffered against extrinsic noise as well as intrinsically stochastic fluctuations in gene expression [113]. In the cell division cycle, the timing of this transition is governed by the activity of cyclin-dependent kinases (CDKs), whose activity is rate-limiting for Rb inactivation. Hence, in both cell division and circadian cycles, stability is conferred by feedback loops whose components are the target for the activity of kinases that determine the rate of progression.

As will be discussed below, both post-translational and TTFL-based models for circadian rhythm generation are consistent with the vast majority of published observations, but importantly, they can be distinguished experimentally. In either case, the key processes that determine periodicity occur post-translationally, either as a delay timer (canonical TTFL model, Figure 1b) or as a self-sustained enzymatic oscillation (post-translational model, Figure 1c). In the next section, we will consider the range of cellular processes that contribute to circadian period determination.

## What is an essential post-translational clock mechanism?

From acetylation to sumoylation, from glycosylation to cysteine oxidation, all the major classes of post-translational protein modification have been implicated as regulating and/or being regulated by circadian timekeeping [114, 115, 116, 117, 118, 119]. Moreover, the activity of many essential cellular systems, such as cAMP/Ca^2+^ signalling [120], the actin cytoskeleton [35], mitochondrial metabolism [121], ion transport [122^•^], redox balance [32,107], and even global protein synthesis rates [38,123], exhibit circadian regulation *in vivo* and *in vitro*, with several also feeding back to affect the amplitude or phase of clock gene expression rhythms, and in some cases even the period of oscillation. If we consider each such system as being an essential timekeeping mechanism, then the cell itself becomes the simplest level of abstraction at which circadian rhythm generation can be understood. This complexity highlights an obstacle for the complete understanding of the mammalian cellular clock, due to inherent difficulties of distinguishing cause from effect in a cycle where oscillations of gene expression are the principal experimental reporter of rhythmicity.

For simplicity’s sake, we will consider redox balance as a point in case, specifically the major cellular redox couples NADH:NAD^+^, NADPH:NADP^+^ and GSH:GSSG. Flux through the oxidative pentose phosphate pathway generates NADPH, providing the primary source of cellular reducing equivalents (NADPH:NADP^+^ usually >100:1 in cytosol) for biosynthesis and maintenance of redox homeostasis (GSH:GSSG usually >100:1 in cytosol); whereas glycolytic activity generates NADH and along with lactate dehydrogenase, is the principle determinant of cytosolic NADH:NAD^+^ ratio (usually <1:100) [124,125]. Glucose catabolism and mitochondrial respiration are circadian regulated in many different contexts *in vivo* and *in vitro* [107,121,126, 127, 128, 129]. In such cases the rate at which cytosolic reducing equivalents (NADPH, NADH) are produced must necessarily also be circadian. If the rate of cytosolic NADH or NADPH oxidation does not match the rate of NAD^+^ or NADP^+^ reduction over the circadian cycle, or if the rate of *de novo* NAD^+^/NADP^+^ synthesis approaches the rate of redox recycling, this must inevitably generate a circadian rhythm in the respective steady state redox couple; and this latter has also been observed in several contexts [121,130, 131, 132, 133]. Similarly, if the rate at which cellular H_2_O_2_ oxidises cytosolic cysteine residues exceeds the rate at which they are reduced by the cell’s antioxidant systems over the daily cycle, then inter- and intramolecular disulphides will form and the cytosolic GSH:GSSG couple will inevitably change over the 24 hour cycle [32,134,135].

Separately, there are multiple established mechanisms by which acute changes in each of these three redox couples have been demonstrated to affect protein function, including transcription factor activity [32,136]. Moreover, there exists ample evidence that acute changes in each of these redox couples are competent to affect the phase and amplitude of circadian gene expression cycles [107,134,137^•^,^138^,^139^, ^140^, ^141^]. If oscillations in redox balance can be a rhythmic output, and can also regulate circadian gene expression rhythms, it is reasonable to suggest that dynamic regulation of redox balance constitutes a central timekeeping mechanism [134,141, 142, 143]. However, when cells are subject to chronic oxidative or reductive stresses, or when cytosolic NADH or NADPH production are pharmacologically inhibited, so long as cells remain viable then circadian rhythms of clock protein production are sustained — with reduced amplitude but with quite modest effects (<10%) on the period of oscillation [107]. Even the activity of important and ubiquitous antioxidant proteins such as peroxiredoxins is not essential for circadian rhythms [82]. This suggests that whilst any circadian regulation of redox balance may well feedback to affect the phase and amplitude of clock gene expression under physiological conditions, it cannot be an essential timekeeping mechanism [107]. Most likely, the balance of any given redox couple simply needs to reside within some permissive range for timekeeping to persist.

Extending this line of argument, it seems likely that acute or sustained genetic or pharmacological perturbation of very many different cellular systems can elicit effects upon the fidelity of circadian timekeeping, because their activity may be permissive for circadian rhythms to be observed, without necessarily meaning that they play any physiological role in period determination or rhythm generation. With reference to beautiful experiments performed with *Neurospora crassa* [144], an essential timekeeping mechanism may be described as a cellular process whose activity contributes to period determination and whose rhythmic regulation is absolutely required for any other circadian rhythm to be observed. Testing essentiality therefore, simply requires that activity be clamped at what would be its average level over the course of a normal circadian cycle. This type of experiment has demonstrated that rhythmic regulation of BMAL1 abundance is not essential for timekeeping at the cellular level, for example [26,145]. Similarly, such experiments allow us to discount the possibility that TOR-mediated rhythmic regulation of global protein synthesis rates constitutes an essential timekeeping mechanism; clearly rhythmic TOR activity is likely to be of enormous physiological relevance and does appear to contribute to circadian timekeeping in healthy cells, but when circadian regulation of TOR activity is abolished, either genetically or pharmacologically, circadian rhythms in clock protein activity persist, albeit with reduced amplitude and modestly lengthened period [146,147^•^,^146–148^].

## Candidates for the post-translational clock mechanism

Applying the above criterion to the many post-translational mechanisms that have been implicated in regulation of circadian rhythms in mammalian cells, we are left with very few plausible candidates for essential post-translational clock mechanisms: casein kinase I (CK1δ/ε) [77,149, 150, 151, 152, 153], casein kinase II (CK2α/α’ catalytic subunit, CK2β regulatory subunit) [154,155], glycogen synthase kinase (GSK3α/β) [156, 157, 158, 159, 160, 161], protein phosphatase 1 (PP1CA/PP1CB/PP1CC catalytic subunits, many regulatory subunits) [149], as well as the ubiquitin-mediated proteasomal degradation system [64,76,169, 170, 171,80,162, 163, 164, 165, 166, 167, 168]. This list is unlikely to be comprehensive, as many post-translational processes have not been fully scrutinised, nor is the evidence for each equally strong. However, each candidate has been implicated independently and multiple times, by both genetic and pharmacological approaches, and all of them act directly to determine the activity of multiple clock protein transcription factors [149,164,168,169]. The functional contribution to cellular timekeeping made by the ubiquitous enzymes mentioned above has largely been interpreted in the context of net increases in site-specific clock protein phosphorylation occurring synergistically over the circadian cycle, with some phosphorylation events promoting nuclear entry [156,172, 173, 174], others regulating transcriptional or translational activity [98,175, 176, 177, 178, 179, 180, 181], and others promoting protein hyperphosphorylation which licenses ubiquitin-mediated proteasomal degradation [167,171,182]. In this context, many other enzymes that post-translationally modify clock proteins, such as AMPK [183,184], *O*-GlcNAc transferase [185,186], and PKA [120,187,188], appear to modulate clock protein activity to communicate temporal change in the local metabolic or extracellular environment but are non-essential for timekeeping function. It is noteworthy that, whilst the activity of each enzyme system listed above is encoded by 2 or more paralogs, overall the activity of each is essential for cellular viability [189, 190, 191].

## Evolutionary implications

In positing a post-translational model for circadian rhythm generation, it is informative to consider the implications for the evolutionary origin of 24-hour timekeeping. Each of the essential post-translational timekeeping mechanisms discussed above is strongly implicated in the regulation of circadian rhythms in the fungal and green lineages, functioning similarly to determine the activity of the clock proteins in other eukaryotic kingdoms despite their transcription factor substrates not being thought to share a common origin [30,192^•^]. Thus, a primarily post-translational cellular clock mechanism suggests a divergent evolutionary history because critical post-translational determinants of 24-hour periodicity are highly conserved (proteasomes, casein kinases, glycogen synthase kinases and protein phosphatases), whilst the identities of TTFL components are poorly conserved. Again, there are clear parallels between post-translational models of circadian timekeeping and the cell cycle. Whilst the CDK enzymatic substrates vary between kingdoms of eukaryotes, the central dogma of eukaryotic cell division is that cell cycle progression is controlled by the activity of the CDKs [110,193], reflecting their common origin in an early eukaryotic progenitor. In contrast, current TTFL-based models for circadian rhythm generation suggest that daily timekeeping has arisen multiple times, convergently, because sequence homology is so poor between the central transcriptional components of the metazoan, fungal and plant circadian clocks [30].

## Circadian timekeeping in the absence of transcription

In TTFL-based models of circadian rhythms, the period of oscillation should be highly sensitive to the rate and timing of transcriptional activation. However, the period of circadian rhythms in mammalian cells is surprisingly resilient against inhibition of RNA polymerase II [194] and, under these conditions, remains compensated against changes in temperature. Moreover, both transcription and translation are temperature-dependent processes [195, 196, 197, 198], whereas circadian period determination is not. A potential solution to this quandary is vested in some recent work where the activity of CK1 against its clock protein substrates was shown to be intrinsically temperature-compensated [199,200^•^], suggesting that since temperature compensation of circadian period occurs post-translationally, then perhaps so too does period determination.

This notion is supported by the precedent set by the entirely post-translational circadian clock encoded by the *KaiBC* and *KaiA* operons in the cyanobacterium *Synechococcus elongatus*. Here the temperature-compensated ∼24 hour rhythm of KaiC protein phosphorylation and complex formation with KaiA/B that occurs in living cells, and normally interacts reciprocally with genome-wide transcriptional regulation, can be reconstituted *in vitro* using just the three recombinant proteins (KaiA, B & C) with Mg.ATP [201]. The remarkable elucidation of the cyanobacterial circadian clock over the last two decades has been reviewed extensively elsewhere [202, 203, 204], and so just a few key similarities with mammalian post-translational timekeeping mechanisms will be considered here. Firstly, KaiC is a slow and inefficient Ser/Thr phosphotransferase, with turnover rates (k_cat_) of the order of h^−1^ [205]; this has some similarity with CK1δ in that the initial phosphorylation of PER peptide substrates has a surprisingly slow k_cat_ (order of m^−1^, [206]) compared with most cellular kinases (usually order of ≥10 s^−1^, [207, 208, 209]). Secondly, the TTFL in cyanobacteria is not sufficient to generate circadian rhythms of gene expression, whereas the post-translational KaiC-based oscillation is [210]. Critically though, when the capacity for transcriptional feedback repression is removed, cellular oscillations in gene expression become less robust, that is the post-translational oscillation is less effective when transcriptional feedback is removed [210]. Finally, KaiC exists primarily in large macromolecular protein complexes with several substrate effectors [211] where dynamic subunit exchange is critical to timekeeping competence [212,213]. This is also mirrored by CK1δ/ε, which has multiple cellular substrates and functions, but consistently co-purifies from the cytosol with the PER and CRY proteins in large (∼1 M Da) macromolecular protein complexes [45^•^].

The successful delineation of the post-translational mechanisms that drive this prokaryotic clockwork raises the question of whether there is evidence for circadian rhythms in the absence of transcription in mammalian and other eukaryotic cells. This question is particularly pertinent given the evolutionary conservation of post-translational timekeeping mechanisms in eukaryotes. The idea of a eukaryotic circadian post-translational oscillator is rendered more plausible by comparison to an analogous mechanism regulating the cell cycle. As mentioned above, the pan-eukaryotic cyclin-dependent kinases (CDKs) are key post-translational enzymes regulating the timing of the G1-S transition of the cell cycle [110,193,214]. Early embryonic cells have a simplified system that alternates between S phase and mitosis, and the activity of CDK1-cyclin complex (originally named in *Xenopus laevis* as maturation promoting factor, MPF) determines the timing of progression to M-phase [215]. Oscillations in MPF were observed in enucleated *Xenopus laevis* eggs, hence driven independently of transcription, and this is now known to result from a negative feedback loop where anaphase-promoting complex (APC/C), acts in concert with kinase/phosphatase positive feedback loops that contribute to stability [216, 217, 218, 219, 220]. The purpose of this analogy is to show that eukaryotic systems can harness post-translational mechanisms to drive oscillations that coordinate diverse cellular processes, using phosphorylation to convey temporal information to many downstream targets including transcription factors, whose change in activity also feeds back to affect the activity of components that drive the oscillation for example by changes in cyclin levels.

To our knowledge, there exist five unrelated experimental observations, most having been independently reproduced, demonstrating that a eukaryotic cellular circadian timekeeping mechanism continues to function in the absence of transcriptional feedback repression. Firstly, in the macroscopic alga *Acetabularia mediterranea*, circadian rhythms of chloroplast migration persist under constant conditions over many days when the cell nucleus is removed, thus rendering nascent nuclear mRNA production impossible [221,222]. Second, in the eukaryotic red alga, *Cyanidioschyzon merolae*, circadian rhythms of protein phosphorylation persist in the absence of translation [223]. Third, an RNA-Seq study revealed circadian rhythms of bioluminescence and photosynthesis in *Lingulodinium polyedrum* do not require rhythmic changes in RNA abundance [224]. Fourth, in the picoeukaryotic alga *Ostreococcus tauri*, rhythms in ion transport and oxidised peroxiredoxin proteins (PRX-SO_2/3_) oscillate with ∼24 hour period under constant darkness — when all RNA production ceases [122^•^,^225^,^226^]. Finally, entrainable temperature-compensated ∼24 hour rhythms of NAD(P)H concentration, PRX-SO_2/3_ abundance and membrane physiology have been observed in isolated mammalian red blood cells which, lacking nuclei and all other organelles, lack the capacity for TTFL-dependent circadian timekeeping [132,227,228^•^].

Because the biological oscillators that drive these diverse non-transcriptional rhythms are not known, it is currently unclear whether these examples represent atypical cellular specialisations that allow circadian rhythms to persist in the absence of transcription, or whether they result from the same post-translational mechanism that confers circadian periodicity upon clock protein activity in other eukaryotic cellular contexts. Given the evolutionarily conserved role of CK1 in determining circadian period and its putative role in temperature compensation, it would be informative to learn whether period and temperature compensation of these non-transcriptional circadian rhythms depend on the activity of CK1, as well as CK2, GSK3, and other conserved post-translational mechanisms that are already strongly implicated in rhythm generation and period determination where transcriptional cycles are present.

## Experimental testing of models for circadian rhythm generation

In light of all the evidence of which we are aware, there exist two plausible models for circadian rhythm generation:

1) **Convergence:** Circadian rhythms have evolved independently at least three times in eukaryotes. In each case these have converged upon a timekeeping mechanism based on transcriptional feedback repression that has recruited the same set of ubiquitous post-translational regulatory mechanisms which function as delay timers to fine-tune gene expression rhythms to a 24 hour period. Cellular circadian rhythms that do not involve cycles of transcriptional repression reflect recent adaptions to specific environmental or physiological niches. The relative timing of clock gene transcriptional activation and repression is the ultimate arbiter of period determination and rhythm generation in eukaryotic organisms.
2) **Divergence:** A post-translational biological oscillation, involving ubiquitous and essential enzymes such as CK1, was present in the last eukaryotic common ancestor. Its targets and function subsequently diverged, recruiting lineage-specific transcriptional effectors (‘clock proteins’) as different kingdoms and phyla arose. Transcriptional feedback repression facilitates hysteresis, thus providing an adaptive advantage that was selected for as it confers rhythmic robustness. Post-translational mechanisms retain executive control of period determination and rhythm generation in modern organisms, but their activity is modulated by their expression level and interacting partners.

There are several experiments that would distinguish between these two models:

1) **Constitutive expression of clock genes:** if the mRNAs of all transcription factors implicated as being essential to circadian regulation were expressed constitutively around the mean of their habitual level of oscillation then no circadian rhythm in any cellular process would be observed according to the convergence model.
2) **Constitutive activation of post-translational regulators:** if the activity of the evolutionarily conserved kinases that determine the activity of clock proteins were clamped around the mean of their habitual level (e.g. by mutation of the auto-phosphorylated regulatory motif of CK1), this would abolish circadian rhythms according to the divergence model
3) **Reconstitution:** if the minimal set of enzymatic components that confer circadian periodicity upon clock protein activity in mammalian cells were known, then an oscillation could be reconstituted in solution according to the divergence model. Conversely the mammalian TTFL will produce circadian rhythms when introduced to an appropriate cellular system that does not normally exhibit circadian timekeeping according to the convergence model.

Should these experiments produce ambiguous results, a third model must be considered: that circadian rhythms are an emergent property of mammalian cells, and whilst the molecular mechanisms of individual components can be dissected, the biological oscillation itself cannot be meaningfully engaged with at any simpler level of abstraction than the entire cell.

## Conclusion

The biological significance of mammalian circadian rhythms can only be properly understood in an organismal context, where the clock in every cell is synchronised with its neighbours, other tissues, and the outside world by myriad humoral timing cues. To fully realise and exploit the potential translational consequences of our innate clockwork it is imperative that we also understand how our cells keep time. Circadian timekeeping occurs cell-autonomously and so, as with the cell division cycle, there is clear utility in the application of cell culture and comparative models for delineating the biochemical mechanisms that allow individual cells to maintain this cell-autonomous oscillation, removed from the continual barrage of extracellular cues that cells experience *in vivo*. Whilst many important components of the mammalian cellular circadian timekeeping machinery have been identified, as well as their interacting partners and functional consequences, the fundamental molecular mechanisms generating the daily oscillation in most cells of the human body are not understood at the same level of detail that allow us to describe the timing of the eukaryotic cell division cycle or the cyanobacterial circadian clock. In the latter two cases, timing function ultimately resides post-translationally, vested in the activity of evolutionarily ancient serine/threonine kinases. Time will tell whether the same is true for eukaryotic circadian clocks.

## Conflict of interest statement

Nothing declared.

## Funding

JSO is supported by the Medical Research Council (MC_UP_1201/4). DCSW is supported by the MRC Doctoral Training Programme and the Frank Edward Elmore Fund.

## References and recommended reading

Papers of particular interest, published within the period of review, have been highlighted as:

• of special interest

## Footnotes

1 We note several excellent studies revealing that post-transcriptional regulation of RNA processing and small interfering RNA-mediated RNA silencing have also been strongly implicated in circadian control, as well as both global and specific regulation of protein synthesis rates [38,98,229, 230, 231, 232, 233, 234, 235, 236]. Whilst perturbation of these essential cellular processes clearly affects circadian gene expression rhythms, to our knowledge it has not been demonstrated that their rhythmic regulation is essential for timekeeping competence and we thus direct the interested reader to these excellent reviews [237, 238, 239].
